# Mendelian randomization**—**a powerful tool to study the causal effects of atrial fibrillation on loss of brain volume

**DOI:** 10.1186/s12916-021-01946-1

**Published:** 2021-03-08

**Authors:** Laura Andreasen

**Affiliations:** 1 Laboratory for Molecular Cardiology, Department of Cardiology, Copenhagen University Hospital – Rigshospitalet, Copenhagen, Denmark; 2 Department of Biomedical Sciences, University of Copenhagen, Copenhagen, Denmark

## Background

Atrial fibrillation (AF) affects a growing proportion of the population, especially the elderly, and has previously been proven to increase the risk of cognitive impairment and dementia independently of stroke [1]. Previous studies have associated AF with loss of brain volume and the association appears to increase with age [2, 3]. The underlying pathophysiological mechanisms between AF, brain volume loss, and cognitive impairment, however, remain unknown. Park et al. [4] sought to investigate the causal effect of AF on brain volume loss using data from genome-wide association studies (GWAS) in a Mendelian randomization (MR) analysis setup.

## Mendelian randomization

MR is a method to elucidate the causal relationship between one phenotype (the exposure) and another (the outcome). The MR method has been in use for decades and relies on the random allocation of parental alleles during meiosis, independently of environmental exposures. MR exploits the assumed random allele assignment, resulting in an unconfounded genotype distribution in a population. As an add-on to classical epidemiological studies, the MR method allows for strong control of confounding and reverse causation, making it a popular tool for genetic epidemiological studies [5]. Today, MR is widely used as a follow-up analysis in GWAS, where millions of common genetic variants, i.e. single nucleotide polymorphisms (SNPs), are tested for association with the trait of interest in a classical case-control study setup. GWAS summary statistics, often publicly available, summarizes results from association testing and are ideal to identify the genome-wide significant genetic variants that are used as instrumental variables for MR.

## Causal effects of atrial fibrillation on lower white matter volume

Using summary data from one of the newest GWAS on AF by Roselli et al., Park and colleagues found evidence of causal effects of AF on lower white matter volume assessed by brain MRI, using the fixed-effects inverse variance weighted MR method. No effect could be found with regard to grey matter volume. The authors also performed a multivariable MR analysis, with the causal estimates of AF on white or grey matter volume adjusted for the effect of stroke, using summary data from the newest stroke GWAS on more than half a million individuals performed by Malik et al. In this case, too, the causal estimates of AF were significant on lower white matter volume and non-significant on lower grey matter volume.

The findings from Park et al. are interesting as they add genetic support to the emerging evidence of a stroke-independent association between AF and brain volume loss, related to both white matter and grey matter loss, as previously shown in observational studies by Moazzami et al. and Stefansdottir et al. [2, 3]. Stefansdottir et al. furthermore found an association between AF and cognitive function, with a lower cognitive test score with increasing AF duration [3]. Moazzami et al. and Stefansdottir et al. suggested different mechanisms to explain their findings, such as altered cerebral hypoperfusion caused by changing cardiac outputs, and AF induced microembolisms [2, 3]. It would be of great interest to know the reflections on the plausible mechanisms behind AF and brain volume loss from Park et al., especially with regard to eventually subsequent cognitive dysfunction.

Notable limitations of the Park et al. study are the lack of Bonferroni correction, and the problem with sample overlap between the different GWAS summary statistics used, as mentioned by the authors. To address the latter limitation, the authors perform sensitivity analyses with a modified genetic instrument, including fewer SNPs with an even stricter significance threshold for association with the trait of interest. These analyses support the main findings, indicating that sample overlap is not a major limitation in this study setup.

## Conclusions

In conclusion, Park et al. use the power of MR and large-scale GWAS to show causal effect of AF on brain volume loss, which is consistent with previous epidemiological studies. Future studies are needed in order to elucidate the causal relationship between brain volume loss and cognitive function.

## Data Availability

Not applicable.
